# Heat-related illness symptoms among migrant farmworkers: a systematic literature review and meta-analysis

**DOI:** 10.1186/s12889-026-28106-5

**Published:** 2026-06-24

**Authors:** Lena van Selm, Sarah Williams, Eman Elafef, Helena Marti-Soler, Simona Vecchi, Francesca de’Donato, Manuela De Sario, Ana Requena-Méndez

**Affiliations:** 1 Barcelona Institute for Global Health (ISGlobal), Hospital Clínic, University of Barcelona, Barcelona, Spain; 2 Facultat de Medicina i Ciències de la Salut, Universitat de Barcelona (UB), Barcelona, Spain; 3 Department of Epidemiology, Lazio Regional Health Service/ASL Roma 1, Rome, Italy; 4 Department of Medicine Solna, Karolinska Institutet, Stockholm, Sweden; 5 CIBER de Enfermedades Infecciosas, Centro de Investigación Biomédica en Red de Enfermedades Infecciosas, CIBERINFEC, Instituto de Salud Carlos III, Madrid, Spain

**Keywords:** Heat, Heat related illnesses, Occupational Exposure, Occupational Health, Outdoor Workers, Transients and Migrants, Farmers

## Abstract

**Background:**

Farmworkers are at risk for heat-related illnesses (HRI) due to intensifying heat exposure resulting from climate change. Migrant farmworkers might be at further increased risk due to limited control over workplace conditions and language and cultural barriers. This systematic literature review and meta-analysis aims to assess the global prevalence of self-reported heat-related illnesses among migrant farmworkers.

**Methods:**

We searched five databases for peer-reviewed literature and additional sources to capture grey literature in any language up to October 2024. Studies providing self-reported heat-related symptoms among migrant farmworkers were included. A random effects model was used to calculate the pooled proportion of individual and combined heat-related health outcomes. Separate pooled estimates were included for each symptom as well as the number of symptoms. Risk of bias was assessed using the JBI quality assessment tool.

**Results:**

Seventeen papers were included, including data from 2966 migrant farmworkers. The pooled proportion for having at least one HRI symptom was 52% (95%CI 39–65) and 21% (95%CI 4–44) reported at least three HRI symptoms. Heterogeneity was high across all pooled estimates (I² ranging from 83% to 99%). The most common symptoms were extreme sweating (46%), weakness (45%), and headache (36%). No differences were found between internal and international migrants nor between studies asking about HRI symptoms experienced in a short-term (e.g., the past week) versus longer periods.

**Discussion and conclusion:**

Migrant farmworkers reported a high proportion of HRI symptoms. Standardized HRI symptom questionnaires and physiological monitoring can aid in early detection to prevent more serious health issues, allow comparison across settings, and support interventions to mitigate heat-related occupational health risks.

**Supplementary Information:**

The online version contains supplementary material available at 10.1186/s12889-026-28106-5.

## Background

Climate change is intensifying heat exposure, leading to more frequent, prolonged, and severe heat waves that pose serious risks to human health, ecosystems, and infrastructure [1]. Globally, 70% of the working population is exposed to excessive occupational heat exposure, which leads to 22.85 million injuries and 18,970 deaths each year. The Africa and Americas regions are the most affected, with 7.2% and 6.7% of all occupational injuries being attributable to excessive heat, respectively [2]. 

Excessive heat exposure, in combination with other factors, leads to the risk of heat stress [2]. Occupational heat stress is defined as the circumstances under which a worker’s body accumulates heat due to the combined effects of metabolic heat, environmental factors and clothing worn [3]. Farmworkers are at increased risk of heat stress due to environmental exposures, including temperature, humidity, and direct sun exposure, combined with metabolic heat generation as a result of heavy physical work [4, 5]. Moreover, as a result of climate change, the risk of heat stress in outdoor workers is increasing in many parts of the world [6]. When heat stress is not managed, heat-related illnesses (HRI) can occur including symptoms such as dizziness, headache or nausea. In severe cases, this can progress to heat stroke, which can cause organ damage and death [7, 8]. In the long term, heat stress can lead to chronic kidney disease [9]. Beyond its health effects, occupational heat stress is also related to productivity losses [10]. 

Migrant and informal workers are overrepresented in physically demanding outdoor jobs, including agriculture, with high exposure to occupational heat stress [2]. In 2019, 7.1% of all global international migrant workers were employed in agriculture, encompassing around 12 million people [11]. In 2021–2022, 68% of farmworkers in the US were foreign-born [12]. In 2021, of all seasonal agricultural workers in Europe, 26% were foreign-born. However, as this number does not include migrants permanently working in the sector the actual number is likely to be higher [13, 14]. Especially, when taking into account that in 2020 an estimated 1.6 million people were working informally in agriculture, likely including a high percentage of (undocumented) migrant workers [13]. 

Workers in precarious situations who have lower capacity to adapt are more susceptible to HRI [2]. Factors including irregular migratory status, low education levels and high economic needs, can put migrant in a precarious situation in which they accept low-paid jobs under unfavorable conditions [15]. In both North America and Europe, migrant farmworkers often endure unfavourable working conditions due to fear of losing employment and income [15–17]. They often have limited control over workplace conditions and lack access to knowledge of heat-related risks and occupational safety training [7, 18]. Farmworkers who migrate to a new location often have not fully acclimatized to environmental conditions as proper acclimatization physical exertion in hot weather typically takes 4 to 14 days, increasing their HRI risk [2, 19]. An investigation by the California Division of Occupational Safety and Health (Cal/OSHA) on hospitalization and death as a result of occupational heat exposure found that 80% of cases occurred within the first four days and almost half on the first day of work [20]. 

Heat stress or heat-related health outcomes can be measured or estimated in different ways. Wet-bulb globe temperature (WBGT), which considers temperature, humidity, wind speed, and thermal radiation, is considered one of the most effective indicators for evaluating the risk of heat stress for populations working in excessive heat [2]. However, the WBGT does not take into account the personal factors of each individual worker. A meta-analysis on heat strain among outdoor workers found that an increase in WBGT only explained small fractions of the variance in core temperature, skin temperature and heart rate and suggested that other factors could be explained by personal factors, including age, sex and medical conditions [21]. Therefore, a more accurate way to measure heat stress in individuals is by using sensors to record physiological measures, including body temperature and/or heart rate [22]. The aforementioned measures quantify heat stress, an intermediary condition that precedes and may lead to HRI but is not synonymous with it [2]. One way to assess HRI is by analysing clinical records, however, morbidity arising from excessive heat exposure at work is under-reported [2]. Another way is using heat-related symptom questionnaires as a potentially cost-effective way to measure HRI in an occupational setting [23]. Therefore, In the absence of clinical data, the use of questionnaires allows to capture HRI symptoms to estimate the burden of HRI among migrant agricultural workers, including less severe symptoms that would not appear in clinical records.

To the best of our knowledge, no meta-analytic estimates of heat-related symptoms among migrant workers are currently available in the literature. In a recent scoping review conducted by the authors of this paper, we mapped the available evidence on self-reported HRI symptoms among migrant farmworkers and identified a body of literature that could facilitate a further quantitative synthesis [24]. Considering the specific marginalized population of interest, which faces substantial barriers to accessing healthcare [24], and the well-documented misclassification of HRI in hospital and emergency department records [25], self-reported symptoms can provide valuable insights into the burden of heat and HRI among migrant farmworkers. The current systematic literature review and meta-analysis aimed to assess the global prevalence of self-reported HRI among migrant farmworkers, providing insights into symptom-based health risk indicators, which could be used to inform occupational policies and interventions for a workforce population recognized as being most at risk.

## Methods

A protocol for this systematic literature review was registered on PROSPERO (ID: CRD42024587002). Findings were reported complying with the Preferred Reporting Items for Systematic Review and Meta-analysis Protocols (PRISMA) 2020 guidelines (Annex 1) [26]. 

### Eligibility criteria

We included quantitative studies with any observational design (e.g., cross-sectional, cohort, or case-control) and quantitative data from mixed-methods studies. Qualitative studies, laboratory studies, modeling studies, case reports, consensus statements, reviews, and meta-analyses, editorials, conference proceedings, letters, books, or opinions were excluded. Studies reporting the proportion of HRI symptoms among farmworkers that were migrants or belonged to ethnic minorities irrespective of the legal status or age, were eligible for inclusion. In this study farmworker refers to those growing and producing agricultural products; warehouse workers were not considered farmworkers. The International Organization for Migration (IOM) definition of migrant was applied: “a person who moves away from his or her place of usual residence, whether within a country or across an international border, temporarily or permanently, and for a variety of reasons.” [27] We differentiated between two migrant categories: international migrants were defined as those who crossed a national border to work abroad, while internal migrant workers were those who relocated to a different region within their country of residence. Studies were only included if either the full sample consisted of migrant farmworkers (international, internal or both) or if disaggregated data were available. In the search strategy, we also applied search terms focusing on ethnic minorities, but no studies on these population were identified (Annex 2).

### Search strategy

Five electronic databases were searched on 1 October 2024: PubMed, Embase, Web of Science, CINAHL and PsycInfo. Studies in any language and from any year of publication were included. On 21 October 2024 the grey literature databases OpenGrey and Climate Change and Human Health Literature Portal were searched as well as the websites of the World Health Organization (WHO), International Organization for Migration (IOM), International Labour organization (ILO), Food and Agriculture Organization (FAO), and World Bank for relevant reports. Finally, the references of all records were screened in three rounds of backwards and one round of forward citation checking.

A broad search strategy was applied, focussing on three concepts: (a) heat exposure, (b) workers, (c) migrants and ethnic minorities. Annex 2 includes the full search strategy for PubMed, which was adapted for each database according to its specific syntax and indexing terms.

After removing duplicates using Endnote version 21, two reviewers (LvS and SW) independently used Rayyan to first screen the titles and abstracts of the identified records for full text assessment and then the full texts of the selected records [28]. Disagreements over inclusion/exclusion at any stage were resolved by discussion between the two reviewers, with the involvement of a third reviewer (MDS) when necessary. Studies which did not meet the inclusion criteria were excluded and the reasons for exclusion were documented.

### Data extraction

Two reviewers (LvS and SW) extracted relevant data using a pre-defined extraction sheet. Extracted data contained study and publication details (location, year of study, study design, study population) as well as either the number or the type of heat-related symptoms. All discrepancies were resolved by consensus with input from MDS. Thirteen authors were approached by email to request additional information of which three eventually shared data that was included in the study.

### Quality assessment

Two independent authors (LvS and EE) independently appraised the quality of studies using a modified version of Joanna–Briggs Institute (JBI) checklist for prevalence studies [29]. The JBI checklist contains a total of nine assessment criteria with four standard answering options (yes/no/unclear/not applicable). (Annex 4). Criteria related to sample selection, sample size, response rate and methods for identification and measurement of the condition were considered most relevant and given double weight, resulting in a maximum score of 14. Studies scoring below 8 were classified as having low methodological quality. The quality assessment results informed both the sensitivity analysis, only including studies with a score of 8 and higher, and the discussion.

### Data analysis

The primary outcome was the pooled proportion of individuals with at least one or a combination of at least three heat-related symptoms, calculated using a proportional meta-analysis via a random effects model using the Metaprop function in R, applying the Freeman-Tukey double arcsine transformation to stabilize variances prior to pooling and back-transforming for reporting. Separate pooled estimates were calculated for the number of symptoms (≥ 3) and per symptom, including only studies that assessed that specific symptom; no harmonization of outcomes or categorization of symptoms (e.g., by severity) was applied across symptoms. Each outcome was reported with the corresponding confidence interval, a measure of precision, and the prediction interval (PI), that reflects the dispersion in effects. We assessed heterogeneity between studies using forest plots and the I^2^ statistic. Because the included studies varied in the recall periods used for self-reported symptoms, ranging from the current day to ever, we categorized them into two groups: short-term recall (covering the current working day up to the past week) and long-term recall (covering any period longer than one week). Differences in pooled proportions between internal and international migrants, and between short- and long-term recall periods, were assessed using the Q heterogeneity chi-squared test for subgroup differences. To assure confidence in the results of the meta-analyses, we included a sensitivity analysis only including studies with high methodological quality. All analyses were performed using R Core Team (version 4.5.0) [30] and meta package (version 8.2-1) [31].

## Results

**Fig. 1:**
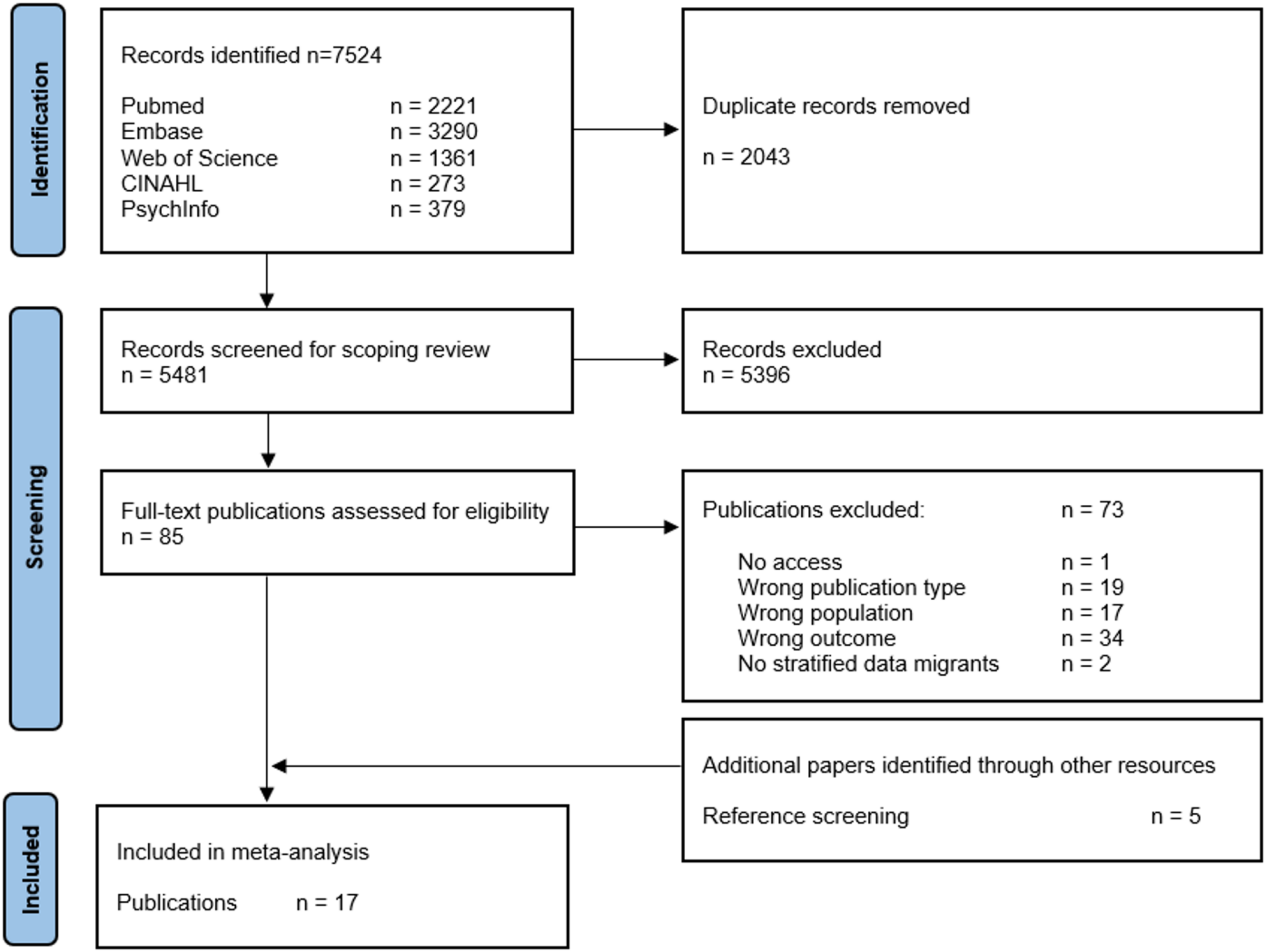
Results from database search and studies included

A total of 5481 unique records were identified of which 5396 were excluded after title and abstract screening. The full text of the remaining 85 publications were assessed, supplemented by five full texts identified via other sources. Finally, 17 studies including data from 2966 migrant farmworkers were included for the meta-analysis (Fig. 1). All studies included self-reported HRI symptoms and no studies with clinical HRI data for migrant agricultural workers were identified. None of the included studies used a validated HRI symptom questionnaire. Table 1 includes the characteristics of the included studies. Sex and age data were only available for the complete samples of the original studies and not for the sub-samples of migrants included in this meta-analysis. Therefore, both the sample size of the original study and the size of the sample used in this meta-analysis are included in Table 1, as well as the sex and age data for the complete sample to serve as an indication for the sub-sample.

**Table 1:** Overview of included studies

#	Reference	Country	Study design	Sample size original study	Sample size meta-analysis^A^	Sex^B^ (% male)	Age^B^ (in years)	Heat metric	Recall period symptoms	Migrant type^C^
1	Albu, I., 2023 [32]	USA	Cohort	115	99^D^	23%	Median: 38.9	Mean heat index: 34.4°C^F^	During that workday	International
2	Arcury, T. A., 2015 [33]	USA	Cross-sectional	101	101	100%	35–44: 36.6% 45+: 27.7%	96.8% days > 26.7 °C 61.2% days > 32.3 °C	In previous 3 months	International and internal
3	Arcury, T. A., 2020 [34]	USA	Cross-sectional	202	63	62.4%	10–13: 21.3%, 14–15: 31,7%, 16–17: 47%	Average temp North Carolina May-Sept 2017: 22.94°C^G^	In the past year	International and internal
4	Bethel, J.W., 2014 [35]	USA	Cross-sectional	100	100	60%	Mean: 31.8 ± 10.1	Daily mean temp: 28.3 °C	In the past week	Internal
5	Boonruksa, P., 2020 [36]	Thailand	Case control	90	90	58.9%	Mean: 42.0 ± 11.0	WBGT mean: 30.6 °C	During the past season	Internal
6	Culp, K., 2019 [37]	USA	Cross-sectional	148	148	100%	18–34: 43.2% 35+: 57.4%	<24 °C WBGT: 26.3% 24–26.9 °C WBGT: 26.3% > 27 °C WBGT: 47.4%^H^	During that workday	International
7	Fleischer, N. L., 2013 [38]	USA	Cross-sectional	405	402	80.5%	Mean: 35.8 ± 13.2	Daily mean temp: 35–40 °C Daily max: 42.2 °C	In the past week	Internal
8	Gelaye, K.A., 2021 [39]	Ethiopia	Cross-sectional	950	950	99.3%	Mean: 26 ± 7.8	Daily mean temp in region: 38 °C	In the past week	Internal
9	Kearney, G. D., 2016 [40]	USA	Cross-sectional	158	141	99.4%	Mean: 36.01 ± 10.38	Daily max: 30–35 °C. Max heat index: 47.2 °C	In the past week	International and internal
10	McQueen, S.L., 2012 [41]	USA	Cross-sectional	85	85	91%	Mean: 30.2 ± 10.6	Dry bulb temp: 34.2 °C WBGT: 30.7 °C	In the past week	International
11	Mirabelli, M. C., 2010 [42]	USA	Cross-sectional	281	281	94.7%	18–33: 48.4% 34–65:51.6%	Average temp North Carolina June-Sept: 23.5°C^G^	Ever	Internal
12	Odame, E.A., 2019 [43]	USA	Cross-sectional	425	292^E^	69%	< 40: 63.3% 40+: 36.7%	WBGT mean: 27.3 °C. WBGT max: 30.6 °C	During that workday	Internal
13	Quiller, G., 2017 [44]	USA	Cross-sectional	46	44	84.8%	Mean: 39.1 ± 14.1	WBGT mean: 27.9° C (August) and 21.2 °C (September)	In the past week	International
14	Riccò, M., 2019 [45]	Italy	Cross-sectional	131	11	81.7%	< 40: 37.4% 40+: 62.6%	Average temp Trento/Mattarello March-Sept 2016: 18.8°C^G^	During previous hot season	International
15	Smith, D. J., 2021 [46]	USA	Cross-sectional	60	60	82%	Mean: 28.8 ± 7	Heat index: 29.14° C	During that workday	Internal
16	Spector, J., 2015 [47]	USA	Cross-sectional	97	90	53%	18–34: 34% 35+: 66%	Daily max temp: 25–36.1 °C Heat index: 28.9° C	In the past week	International
17	Villanueva-Gómez, R., 2023 [48]	Canada	Mixed methods	9	9	100%	Mean: 38.2 ± 8.1	Average temp Montreal first week of Sept 2022: 19.3°C^G^	In the current season	International

^A^Studies were only included for analysis if 1) 100% of the sample consisted of international and / or internal migrant workers, 2) the authors provided segregated data for international and / or internal migrant worker groups within the total sample (i.e., studies #3, #13, #14 and #16)

^B^Sex and age are reported from the total sample of the original study, as we did not have access to this data for the sub-sample used in the meta-analysis

^C^Migrant type reflects how studies were included in the meta-analysis, based on the availability of (segregated) data. The studies by Albu *et al*. and Villanueva-Gómez *et al*. explicitly stated that the samples consisted exclusively of international migrants. For the other studies, the absence of a migrant type implies that (segregated) data for this migrant category were not reported

^D^For the analysis data from the sample from August 2020 was used (as the first sample from January 2020 was not collected during the hot season), this data was provided by the authors and is not included in the cited article

^E^Only data from outdoor workers was included for the analysis

^F^This was a longitudinal study with five different points of measurements. The first measurement in summer (august 2020) is reported

^G^No heat matrices provided in paper; measures from National Oceanic and Atmospheric Administration (NOAA) or MeteoStat used to provide mean temperature during study period

^H^only measured for sub-sample of 57 participants

Most studies were conducted in the US (*n* = 13) and the others were from Canada, Ethiopia, Italy and Thailand. Two studies included international migrants exclusively, while the other studies were either a mix between international and internal migrants or included one migrant type without specifying the other (Table 1). Finally, seven studies provided data on international migrants [32, 37, 41, 44, 45, 47, 48], seven on internal migrants [35, 36, 38, 39, 42, 43, 46], and three on both internal and international migrants [33, 34, 40]. All studies used a cross-sectional design, except for one cohort [32] and one case control study [36]. Using the JBI quality assessment tool, five studies were rated as low quality due to unclear or inappropriate sample selection, response rate, and condition definition and measurement. All studies adequately described participants and settings and employed reliable statistical analyses.

**Fig. 2:**
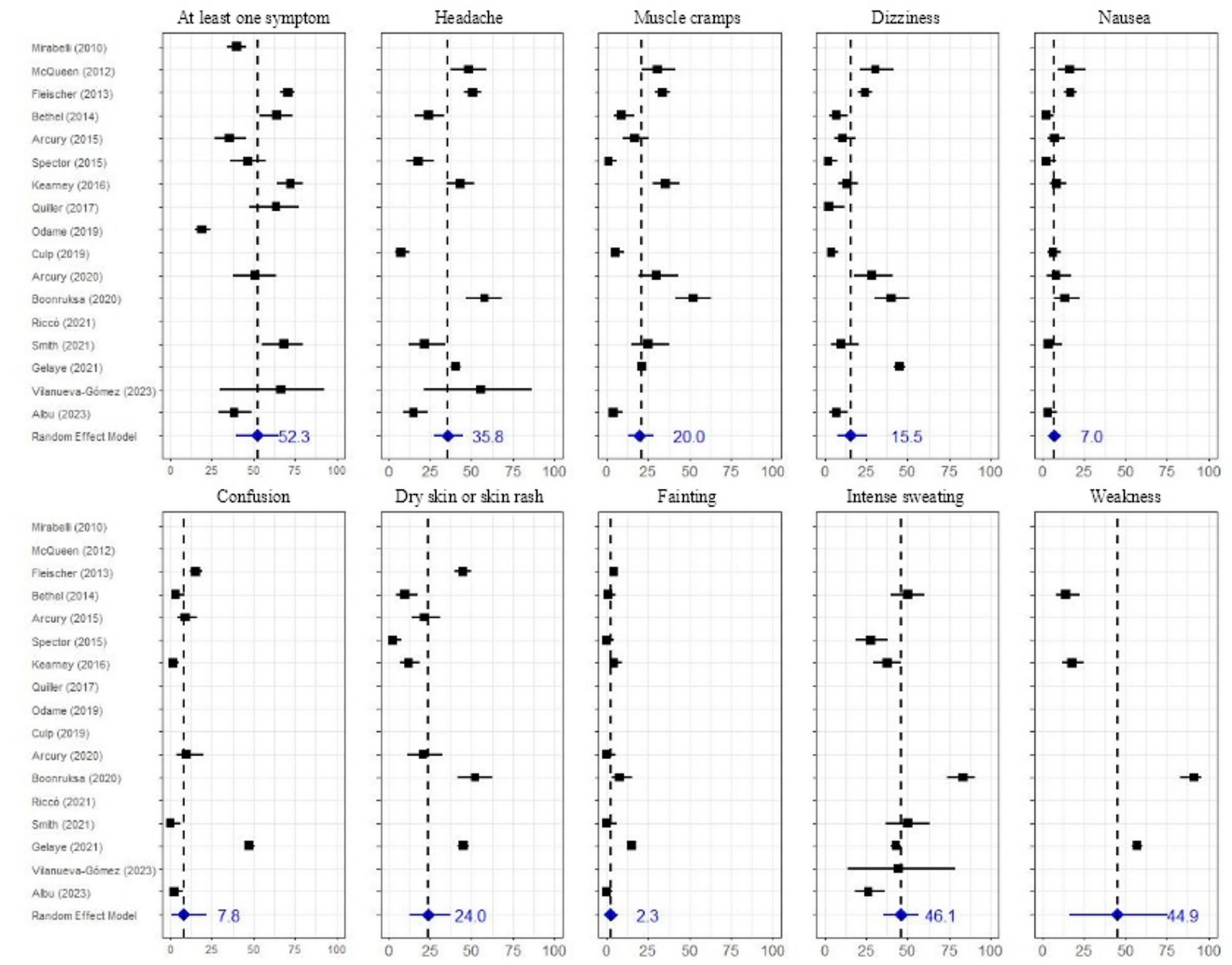
Proportion of HRI symptoms per study including confidence intervals

The pooled proportion of self-reported heat-related symptoms between all studies was 52% (95% CI 39–65; PI 0.09–0.94; I^2^ 96%) for at least one symptom and 21% (95% CI 4–44; PI 0.00–0.97; I^2^ 99%) for at least three symptoms (Fig. 2; Table 2). The questionnaires used in the studies included different numbers and combinations of HRI symptoms (Annex 3). The most prevalent symptoms were extreme sweating (46%; 95% CI 35–57; PI: 0.13–0.81; I^2^ 92%), weakness (45%; 95% CI 16–76; PI: 0.00–1.00; I^2^ 99%), headache (36%; 95% CI 27–45; PI: 0.10–0.67; I^2^ 92%) dry skin or skin rash (24%; 95% CI 13–38; PI: 0.00-0.74; I^2^ 97%) and muscle cramps (20%; 95% CI 13–28; PI: 0.01–0.55; I^2^ 95%). When distinguishing between migrant types, the proportion of workers reporting at least on symptom remained unchanged, with 52% (95% CI 39–65; PI 0.12–0.91; I^2^ 87%) of international migrants and 53% (95% CI 36–69; PI 0.04–0.98; I^2^ 97%) of internal migrants experiencing at least one HRI symptom. Likewise, the pooled proportion of most symptoms did not differ significantly between migrant types, with the exception of heavy sweating and dry skin or skin rash, which was higher among internal migrants (Table 2). Heterogeneity was high across all pooled estimates (I² ranging from 65% to 99%), and pooled proportions should be interpreted alongside the prediction intervals reported, which capture the dispersion of true effects across studies.

**Table 2:** Heat-related symptoms in migrant farmworkers

	Total					International migrants					Internal migrants					
	Sample size	I^2^	Pooled proportion	# Studies	Prediction interval	Sample size	I^2^	Pooled proportion	# Studies	Prediction interval	Sample size	I^2^	Pooled proportion	# Studies	Prediction interval	*p*-value
At least 1 symptom	1682	96%	**0.52** (0.39–0.65)	12	0.09–0.94	522	87%	**0.52** (0.39–0.65)	7	0.12–0.91	1413	97%	**0.53** (0.36–0.69)	8	0.04–0.98	0.9703
At least 3 symptoms	1622	99%	**0.21** (0.04–0.44)	6	0.00-0.97						1512	99%	**0.28** (0.09–0.53)	4	0.00–1.00	
Headache	2026	92%	**0.36** (0.27–0.45)	10	0.10–0.67	424	91%	**0.33** (0.18–0.50)	5	0.00-0.85	1743	90%	**0.40** (0.32–0.49)	6	0.14–0.70	0.3864
Muscle cramps	2329	95%	**0.20** (0.13–0.28)	12	0.01–0.55	702	94%	**0.15** (0.06–0.28)	7	0.00-0.65	1880	91%	**0.27** (0.19–0.35)	8	0.05–0.58	0.2424
Dizziness	2373	97%	**0.15** (0.08–0.26)	13	0.00-0.63	746	86%	**0.10** (0.05–0.17)	8	0.00-0.39	1880	97%	**0.21** (0.11–0.34)	8	0.00-0.70	0.1001
Nausea	1379	83%	**0.07** (0.04–0.11)	11	0.00-0.24	702	65%	**0.06** (0.03–0.10)	7	0.00-0.19	930	81%	**0.08** (0.04–0.13)	7	0.00-0.28	0.5250
Confusion	2064	99%	**0.08** (0.00-0.22)	9	0.00-0.74	527	68%	**0.05** (0.02–0.09)	5	0.00-0.22	1790	99%	**0.09** (0.00-0.27)	7	0.00-0.84	0.4809
Dry skin or skin rash	1937	97%	**0.24** (0.13–0.38)	8	0.00-0.76	370	86%	**0.12** (0.04–0.23)	4	0.00-0.73	1820	96%	**0.29** (0.18–0.42)	7	0.01–0.75	0.0314*
Fainting	1995	94%	**0.02** (0.00-0.07)	9	0.00-0.26	368	67%	**0.01** (0.00-0.03)	4	0.00-0.21	1779	93%	**0.04** (0.01–0.09)	7	0.00-0.29	0.1564
Sweating	1583	92%	**0.46** (0.35–0.57)	9	0.13–0.81	383	66%	**0.35** (0.26–0.45)	5	0.12–0.62	1341	94%	**0.53** (0.39–0.67)	5	0.07–0.96	0.0404*
Weakness	1281	99%	**0.45** (0.16–0.76)	4	0.00–1.00						1281	99%	**0.45** (0.16–0.76)	4	0.00–1.00	

*P*-values calculated using the Q heterogeneity chi-squared test for subgroup differences

*Statistically significant

A sub-analysis was done including only studies from the US and Canada, where results were similar to those of the full sample (Annex 4). As studies varied in the recall period considered when asking about the symptoms experienced, a distinction was made between short-term recall, ranging from the current workday to the past week (e.g., did you have a headache working today´s work shift?), and long-term recall, defined as any period longer than the past week (e.g., have you felt dizzy while working during the previous warm season?). No differences were found in the pooled proportion of any symptoms between studies using short-term versus long-term recall periods (Table 3). At least one symptom was reported by 42% (95% CI 34–50; PI: 0.15–0.72; I^2^ 50%) in the long-term recall group and 55% (95% CI 37–72; PI: 0.04–0.99; I^2^ 97%) in the short-term recall group. The specific symptoms included for both recall periods were muscle cramps (long term: 32%; 95% CI 13–55; PI: 0.00–1.00; I^2^ 93%. Short term: 17%; 95% CI 9–25; PI: 0.00-0.52; I^2^ 95%), dizziness (long term: 25%; 95% CI 9–46; PI: 0.00-0.99; I^2^ 91%. Short term: 13%; 95% CI 4–64; PI: 0.00-0.64; I^2^ 98%), nausea (long term: 9%; 95% CI 6–14; PI: 0.02–0.21; I^2^ 11%. Short term: 6%; 95% CI 3–11; PI: 0.00-0.27; I^2^ 88%) and dry skin or skin rash (31%; 95% CI 13–52; PI: 0.00-0.10; I^2^ 92%. Short term: 20%; 95% CI 7–38; PI: 0.00-0.84; I^2^ 98%) (Table 3).

**Table 3:** Heat-related symptoms assessed over long-term versus short-term recall periods

	Long-term recall					Short-term recall					
	Sample size	I^2^	Pooled proportion	# Studies	Prediction interval	Sample size	I^2^	Pooled proportion	# Studies	Prediction interval	*p*-value
At least 1 symptom	454	50%	**0.42** (0.34–0.50)	4	0.15–0.72	1228	97%	**0.55** (0.37–0.72)	8	0.04–0.99	0.1841
At least 3 symptoms	-	-	-	1	-	1611	99%	**0.21** (0.04–0.46)	5	0.00-0.97	
Headache	-	-	-	2	-	1927	93%	**0.32** (0.24–0.42)	8	0.08–0.64	
Muscle cramps	254	93%	**0.32** (0.13–0.55)	3	0.00–1.00	2075	95%	**0.17** (0.09–0.25)	9	0.00-0.52	0.0751
Dizziness	254	91%	**0.25** (0.09–0.46	3	0.00-0.99	2119	98%	**0.13** (0.04–0.64)	10	0:00–0:64	0.2741
Nausea	254	11%	**0.09** (0.06–0.14)	3	0.02–0.21	1125	88%	**0.06** (0.03–0.11)	8	0.00-0.27	0.3200
Confusion	-	-	-	2	-	1900	99%	**0.07** (0.00-0.24)	7	0.00-0.80	
Dry skin or skin rash	254	92%	**0.31** (0.13–0.52)	3	0.00-0.10	1683	98%	**0.20** (0.07–0.38)	5	0.00-0.84	0.3952
Fainting	-	-	-	2	-	1842	95%	**0.02** (0.00-0.07)	7	0.00-0.29	
Sweating	-	-	-	2	-	1484	77%	**0.40** (0.34–0.47)	7	0.21–0.61	
Weakness	-	-	-	1	-	1191	99%	**0.28** (0.05–0.62)	3	0.00–1.00	

Long term: In the current season – ever

Short term: During the current working day – in the past week

*P*-values calculated using the Q heterogeneity chi-squaredtest for subgroup differences

A sensitivity analysis was performed including only studies that scored ≥ 8 on the JBI prevalence checklist (Annex 5), but no difference was found from the results including all studies (Annex 6). For example, the proportion of headache in the full sample was 35% (95% CI 27–44; PI: 0.09–0.67; I^2^ 92%) versus 30% in the sensitivity analysis (95% CI 21–40; PI: 0.05–0.65; I^2^ 94%) and for muscle cramps 21% in the full sample (95% CI 14–29; PI: 0.01–0.57; I^2^ 94%) versus 16% in the sensitivity analysis (95% CI 10–24; PI: 0.00-0.50; I^2^ 94%).

## Discussion

The findings of this study show that around half of migrant farmworkers included in this analysis experienced at least one heat-related symptom while working. This is the first quantitative synthesis on heat-related occupational health among migrant farmworkers allowed by a growing and consistent body of evidence according to a previous scoping review on the topic [24], while evidence based on clinical records and physiological measures remains limited. As some HRI symptoms are general and non-specific, defining HRI as a cluster of several symptoms could ensure a more robust definition and assessment of heat stress among workers [23]. For example, some studies define a case of HRI as the presence of at least three symptoms [38, 39, 45, 49]. When adopting that definition of HRI, our findings indicate that 21% of the global migrant farmworkers population suffered from HRI. The most prevalent symptoms were: sweating (46%), weakness (45%), headache (35%), dry skin or skin rash (24%), and muscle cramps (21%). No differences were found between international and internal migrants nor between symptoms reported over a longer recall period (over a week) versus a shorter recall period (within the past week). Given the frequently high heterogeneity across studies (I² values often exceeded 90%), these pooled estimates should be interpreted with caution, as they reflect a wide range of underlying prevalence rates across different settings, populations, and symptoms, as further illustrated by the wide prediction intervals reported.

Heat-related symptoms assessed by self-reported questionnaires are subject to bias, primarily recall bias, due to personal characteristics affecting risk perception and symptom reporting. Although validated questionnaires are a mean to provide more accurate symptom assessment, their development and use are particularly challenging in the context of heat-related occupational health among migrant workers. This is mainly due to the lack of a golden standard for heat-related diseases, since clinical records are well known to underestimate the true burden of heat related illness [25], and migrants workers face specific barriers to healthcare services [24]. These aspects limit accurate comparison between studies overall and by specific type of HRI symptoms and interpretation of heterogeneity between studies. Throughout the included studies, only three studies conducted some type of internal validation for their questionnaires. One study mentioned having checked for internal validity and consistency for the translated questionnaire but did not provide further information [39], another reported only having included items with a correlation coefficient > 0.80 [45], and a third used a group of six agricultural workers to evaluate content validity [47]. However, none of them described a process specifically focusing on assessing all potential HRI symptoms and eliminating the ones that showed low internal consistency and reliability. The Heat Illness Symptom Index (HISI), created to detect milder forms of heat illness in athletes, used such an approach and was found to correlate with traditional risk factors for HRI. However, the HISI was not compared to physiological outcomes (e.g., body temperature or heart rate) nor tested in occupational settings [50]. To allow more accurate comparison between settings and giving stronger value to the results, a standardized questionnaire for HRI symptoms needs to be developed and validated in a migrant farmworker population.

Beyond questionnaire design and internal validity, the relationship between self-reported HRI symptoms and physiological indicators or clinical assessments of heat stress remains largely unexplored. While for other occupational diseases, including musculoskeletal and skin disorders, the level of agreement between self-reporting and professional assessment was moderate [51], no previous studies have assessed this relationship for HRI. Among the studies included in our review, three reported physiological data in addition to self-reported HRI symptoms. McQueen (2012) found that 10.6% of migrant farmworkers had at least three HRI symptoms, while estimates of heat stress based on physiological measures ranged from 0% to 33%, depending on whether body temperature, heart rate, or body weight loss was used [41]. However, this analysis only included 18 participants. Boonruksa et al. (2020) reported significant increases in blood pressure, heart rate, and body temperature over the work shift; however, no assessment was made of the proportion of workers meeting criteria for heat stress based on these measures, nor were they compared with self-reported HRI symptoms [36]. Both Boonruksa et al. and Albu et al. (2023) reported levels of pre- and post-shift dehydration [32, 36], but although dehydration is an important clinical indicator associated with occupational heat stress, in the absence of additional physiological measures no conclusions can be drawn on heat stress levels among participants [52]. In conclusion, while HRI symptom questionnaires provide valuable insight into workers’ experiences and may support early identification of heat-related health effects, findings should be interpreted with caution given the limited evidence linking self-reported symptoms to clinical data on heat stress; future research should explicitly examine this relationship in occupational settings.

Multiple factors can make farmworkers more vulnerable to heat related illnesses. Prevention measures and access to healthcare services are essential for limiting the burden of HRI. In this regard, farm management and employer decisions are key for prevention and shape workers´ individual preventive behaviours. Among migrant farmworkers in the US, suboptimal levels of employer-provided prevention were identified; only 60.4% received training on occupational heat, 43.7% acclimatized through gradually increased working hours at the start of the agricultural season, and on hot days, 34.0% adapted working hours and 32.2% modified work activities [24]. Moreover, certain work organisation factors undermine heat-prevention as they provide incentives to work faster and take less breaks. For example, piece-rate payment has been associated with increased work effort, reduced frequency of rest and hydration breaks, and an increased risk of HRI symptoms [47, 53]. In addition, migrant farmworkers reported that supervisors pressured them to work faster by shouting, denying breaks, or threatening to withhold wages [54, 55]. On the other hand, guidelines recommend to modify work schedules to avoid working during the hottest hours of the day [2]. In addition to the management of resting schemes, changes in farm design can protect workers from occupational heat exposure. For example, access to water and shade structures reduce occupational heat exposure and resting in shaded areas has been associated with lower HRI rates [56, 57]. Beyond preventive measures, access to healthcare services is essential when more severe HRI symptoms occur. In the US, in 2022, only 24% of foreign-born migrant farmworkers and 38% of internal migrant farmworkers had health insurance, and uninsured workers were 34% less likely to have used healthcare services in the previous year [12]. Costs was cited as a concern for visiting healthcare services for HRI [58]. In Europe, migrant farmworkers also reported barriers to accessing healthcare services [15]. 

With increasing heat exposure resulting from climate change, occupational heat exposure is expected to rise. Monitoring HRI at the workplace could allow early detection and help prevent more serious health issues. A standardized and validated HRI symptom questionnaire should be developed for this purpose and, where possible, accompanied by physiological monitoring. In addition, some studies have evaluated the impact of both pesticide exposure and heat exposure — measured using the physiological strain index (PSI)) — on farmworker health, and findings suggest that pesticides may act synergistically with heat stress to exacerbate adverse health outcomes, though future studies utilizing physiological monitoring are needed to further investigate this effect [59, 60]. Furthermore, farmers working inside greenhouses during summer can be exposed to temperatures exceeding outdoor temperatures [61], but further evidence needs to clarify its impact on HRI. Finally, HRI studies should also be conducted in other outdoor working populations exposed to occupational heat, including construction workers. Finally, working environments should be fostered in which migrant farmworkers have access to both preventive measures and healthcare services to limit HRI.

### Strengths and limitations

This study is the first to compare and pool the proportion of HRI among migrant farmworkers globally and therefore could serve as a reference point for future studies assessing local contexts. This review had several limitations. First, the evidence presented in all studies is limited to self-reported symptoms, which has the risk of being subjective. However, as many of these symptoms (e.g., headache, dizziness, nausea) are not directly observable or measurable through physiological assessments, this is a widely used way to assess HRI. Second, heterogeneity in the timeframe in which participants were asked having experienced HRI symptoms, ranging from the day of the survey to ever (as shown in Table 1) made comparison less accurate, although we accounted for this by calculating separate values for short- and long-term recall reporting. Third, the review has limited geographical coverage, as the far majority of studies were carried out in the US and therefore findings might not be transferable to other settings with different climates, labour regulations and health systems. This may especially be the case for low-and-middle income countries, where working and living conditions are often more precarious but further research is needed to confirm this. Fourth, we were unable to control for potential confounders such as age, gender and comorbidities as this information was not available for the migrant sub-sample in all studies. As an indication, we included the sex and age of the full sample of the original studies in Table 1. Finally, we were not able to include the temperatures at the study sites in the analysis due to heterogeneity among the heat metrics used to report temperatures. In addition, it would be inaccurate to use the heat metric at the time of the survey as participants were asked about a longer period of time. To include an indication of heat at each study site, Table 1 includes the heat metrics at the time of the survey as reported in the paper or according to data from the National Oceanic and Atmospheric Administration (NOAA) or MeteoStat.

## Conclusions

Migrant farmworkers reported a high proportion of HRI symptoms, the main ones being heavy sweating, weakness, headache, dry skin or skin rash and muscle cramps. Around half of the workers reported having experienced at least one HRI symptom and around one fifth reported at least three symptoms. No differences were found between studies asking about HRI symptoms experienced in a short-term recall period (e.g., the past week) versus a long-term recall period (e.g., throughout the whole season). Occupational studies should develop and adopt a standardized HRI questionnaire and the relation with physiological symptoms should also be assessed.

## Supplementary Information

Supplementary Material 1.

## Data Availability

Most data analyzed in this systematic literature review were obtained from publicly available articles and datasets. In some cases, the authors of the included papers were contacted for additional information or clarifications. Any materials created in this review, such as the data extraction sheet, could be shared by the authors on request.

## Abbreviations

USA: United states of america
HRI: Heat-related illness(es)
WBGT: Wet-bulb globe temperature
Cal/OSHA: California division of occupational safety and health administration
IOM: International organization for migration
PRISMA: Preferred reporting items for systematic review and meta-analysis protocols
JBI: Joanna briggs institute
PI: Prediction interval
WHO: World health organization
ILO: International labour organization
FAO: Food and agriculture organization
USG: Urine specific gravity
HISI: Heat illness symptom index
