# Supplementary material for: Heat-related illness symptoms among migrant farmworkers: a systematic literature review and meta-analysis
Source: BMC Public Health. 2026 Jun 24;26:2353. doi: 10.1186/s12889-026-28106-5 (PMC13471271; doi:10.1186/s12889-026-28106-5)
Supplement: Supplementary file 1 [file 12889_2026_28106_MOESM1_ESM.docx]

**Supplementary materials**

**Heat-Relates Illness among Migrant Farmworkers: A Systematic Literature Review and Meta-Analysis**

**Materials:**

- Annex 1: PRISMA 2020 Checklist
- Annex 2: Full search strategy
- Annex 3: HRI symptoms included in questionnaire per study
- Annex 4: Heat-related illness symptoms in migrant farmworkers – US and Canada only
- Annex 5. Critical appraisal of included studies using the JBL prevalence checklist
- Annex 6: Sensitivity analysis – only studies with a score ≥8 on the JBL prevalence checklist

**Annex 1:**

| **Section and Topic** | **Item #** | **Checklist item** | **Location where item is reported** |
| --- | --- | --- | --- |
| **TITLE** | | |  |
| Title | 1 | Identify the report as a systematic review. | P1 |
| **ABSTRACT** | | |  |
| Abstract | 2 | See the PRISMA 2020 for Abstracts checklist. |  |
| **INTRODUCTION** | | |  |
| Rationale | 3 | Describe the rationale for the review in the context of existing knowledge. | P2-3 |
| Objectives | 4 | Provide an explicit statement of the objective(s) or question(s) the review addresses. | P3 |
| **METHODS** | | |  |
| Eligibility criteria | 5 | Specify the inclusion and exclusion criteria for the review and how studies were grouped for the syntheses. | P4-5 |
| Information sources | 6 | Specify all databases, registers, websites, organisations, reference lists and other sources searched or consulted to identify studies. Specify the date when each source was last searched or consulted. | P5 |
| Search strategy | 7 | Present the full search strategies for all databases, registers and websites, including any filters and limits used. | Annex 2 |
| Selection process | 8 | Specify the methods used to decide whether a study met the inclusion criteria of the review, including how many reviewers screened each record and each report retrieved, whether they worked independently, and if applicable, details of automation tools used in the process. | P5 |
| Data collection process | 9 | Specify the methods used to collect data from reports, including how many reviewers collected data from each report, whether they worked independently, any processes for obtaining or confirming data from study investigators, and if applicable, details of automation tools used in the process. | P5 |
| Data items | 10a | List and define all outcomes for which data were sought. Specify whether all results that were compatible with each outcome domain in each study were sought (e.g. for all measures, time points, analyses), and if not, the methods used to decide which results to collect. | P5 |
|  | 10b | List and define all other variables for which data were sought (e.g. participant and intervention characteristics, funding sources). Describe any assumptions made about any missing or unclear information. | P5 |
| Study risk of bias assessment | 11 | Specify the methods used to assess risk of bias in the included studies, including details of the tool(s) used, how many reviewers assessed each study and whether they worked independently, and if applicable, details of automation tools used in the process. | P5-6 |
| Effect measures | 12 | Specify for each outcome the effect measure(s) (e.g. risk ratio, mean difference) used in the synthesis or presentation of results. | P6 |
| Synthesis methods | 13a | Describe the processes used to decide which studies were eligible for each synthesis (e.g. tabulating the study intervention characteristics and comparing against the planned groups for each synthesis (item #5)). | P6 |
|  | 13b | Describe any methods required to prepare the data for presentation or synthesis, such as handling of missing summary statistics, or data conversions. | NA |
|  | 13c | Describe any methods used to tabulate or visually display results of individual studies and syntheses. | P6 |
|  | 13d | Describe any methods used to synthesize results and provide a rationale for the choice(s). If meta-analysis was performed, describe the model(s), method(s) to identify the presence and extent of statistical heterogeneity, and software package(s) used. | P6 |
|  | 13e | Describe any methods used to explore possible causes of heterogeneity among study results (e.g. subgroup analysis, meta-regression). | P6 |
|  | 13f | Describe any sensitivity analyses conducted to assess robustness of the synthesized results. | P6 |
| Reporting bias assessment | 14 | Describe any methods used to assess risk of bias due to missing results in a synthesis (arising from reporting biases). | NA |
| Certainty assessment | 15 | Describe any methods used to assess certainty (or confidence) in the body of evidence for an outcome. | NA |
| **RESULTS** | | |  |
| Study selection | 16a | Describe the results of the search and selection process, from the number of records identified in the search to the number of studies included in the review, ideally using a flow diagram. | P6,  Figure 1 |
|  | 16b | Cite studies that might appear to meet the inclusion criteria, but which were excluded, and explain why they were excluded. | NA |
| Study characteristics | 17 | Cite each included study and present its characteristics. | Table 1 |
| Risk of bias in studies | 18 | Present assessments of risk of bias for each included study. | Annex 4 |
| Results of individual studies | 19 | For all outcomes, present, for each study: (a) summary statistics for each group (where appropriate) and (b) an effect estimate and its precision (e.g. confidence/credible interval), ideally using structured tables or plots. | Table 2, Table 4, Figure 2 |
| Results of syntheses | 20a | For each synthesis, briefly summarise the characteristics and risk of bias among contributing studies. | P7 |
|  | 20b | Present results of all statistical syntheses conducted. If meta-analysis was done, present for each the summary estimate and its precision (e.g. confidence/credible interval) and measures of statistical heterogeneity. If comparing groups, describe the direction of the effect. | P6-7 |
|  | 20c | Present results of all investigations of possible causes of heterogeneity among study results. | P6-7 |
|  | 20d | Present results of all sensitivity analyses conducted to assess the robustness of the synthesized results. | P7 |
| Reporting biases | 21 | Present assessments of risk of bias due to missing results (arising from reporting biases) for each synthesis assessed. | NA |
| Certainty of evidence | 22 | Present assessments of certainty (or confidence) in the body of evidence for each outcome assessed. | P6-7 |
| **DISCUSSION** | | |  |
| Discussion | 23a | Provide a general interpretation of the results in the context of other evidence. | P8-9 |
|  | 23b | Discuss any limitations of the evidence included in the review. | P9 |
|  | 23c | Discuss any limitations of the review processes used. | P9 |
|  | 23d | Discuss implications of the results for practice, policy, and future research. | P8-10 |
| **OTHER INFORMATION** | | |  |
| Registration and protocol | 24a | Provide registration information for the review, including register name and registration number, or state that the review was not registered. | P4 |
|  | 24b | Indicate where the review protocol can be accessed, or state that a protocol was not prepared. | P4 |
|  | 24c | Describe and explain any amendments to information provided at registration or in the protocol. | NA |
| Support | 25 | Describe sources of financial or non-financial support for the review, and the role of the funders or sponsors in the review. | P10 |
| Competing interests | 26 | Declare any competing interests of review authors. | P10 |
| Availability of data, code and other materials | 27 | Report which of the following are publicly available and where they can be found: template data collection forms; data extracted from included studies; data used for all analyses; analytic code; any other materials used in the review. | P10 |

*From:* Page MJ, McKenzie JE, Bossuyt PM, Boutron I, Hoffmann TC, Mulrow CD, et al. The PRISMA 2020 statement: an updated guideline for reporting systematic reviews. BMJ 2021;372:n71. doi: 10.1136/bmj.n71. This work is licensed under CC BY 4.0. To view a copy of this license, visit <https://creativecommons.org/licenses/by/4.0/>

**Annex 2: full search strategy**

| **#** | **Searches** |
| --- | --- |
| 1 | “heat*”[Title/Abstract] OR “sunstroke”[Title/Abstract] OR “sun stroke” [Title/Abstract:~3] OR “hot weather”[Title/Abstract:~3] OR “hot temperature”[Title/Abstract] OR “hot temperatures”[Title/Abstract:~3] OR “hot climate”[Title/Abstract:~3] OR “hot climates”[Title/Abstract:~3] OR “warm weather”[Title/Abstract:~3] OR “warm temperature”[Title/Abstract:~3] OR “warm temperatures”[Title/Abstract:~3] OR “warm climate”[Title/Abstract:~3] OR “warm climates”[Title/Abstract:~3] OR “WBGT”[Title/Abstract] OR “wet bulb globe temperature”[Title/Abstract] OR “extreme temperature”[Title/Abstract:~3] OR “extreme temperatures”[Title/Abstract:~3] OR “thermal discomfort” [Title/Abstract:~3] OR “temperature* change*”[Title/Abstract] OR “temperature* increase*” [Title/Abstract] OR "Extreme Hot Weather"[Mesh] OR "Heat Stress Disorders"[Mesh] OR "Hot Temperature"[Mesh] |
| 2 | “employ*”[Title/Abstract] OR “labour*”[Title/Abstract] OR “labor*”[Title/Abstract] or “occupation*”[Title/Abstract] or “job”[Title/Abstract] OR “jobs”[Title/Abstract] OR “work*”[Title/Abstract] OR “staff*”[Title/Abstract] OR “farmer*”[Title/Abstract] OR “farmwork*”[Title/Abstract] OR "Occupational Groups"[Mesh] OR "Occupational Exposure"[Mesh] OR "Occupational Health"[Mesh] |
| 3 | “Migra*”[Title/Abstract] OR “immigr*”[Title/Abstract] OR “emigr*”[Title/Abstract] OR “refugee*”[Title/Abstract] OR “asylum*”[Title/Abstract] OR “foreign*”[Title/Abstract] OR “undocument*”[Title/Abstract] OR “non-citizen*”[Title/Abstract] OR “citizenship*”[Title/Abstract] OR “transient worker”[Title/Abstract:~5] OR “displac*”[Title/Abstract] OR “diaspora*”[Title/Abstract] OR “latin*”[Title/Abstract] OR “hispanic*”[Title/Abstract] OR “expat*”[Title/Abstract] OR “nationalit*”[Title/Abstract] OR “non-resident”[Title/Abstract] OR “non-residents”[Title/Abstract] OR “newly-arrived” [Title/Abstract] OR “newcomer*”[Title/Abstract] OR “new-comer*”[Title/Abstract] OR “foreign-born”[Title/Abstract] OR “country origin”[Title/Abstract:~3] OR “country birth”[Title/Abstract:~3] OR “place birth”[Title/Abstract:~3] OR “birthplace”[Title/Abstract] OR “refugi*”[Title/Abstract] OR “asile”[Title/Abstract] OR “etrang*”[Title/Abstract] OR “sans-papiers” [Title/Abstract] OR “transitoire*”[Title/Abstract] OR “citoyen*”[Title/Abstract] OR “Transients and Migrants”[Mesh] OR “Refugees”[Mesh] OR “Emigrants and Immigrants”[Mesh]  OR “Ethnic and Racial Minorities"[MeSH] OR "Ethnicity"[MeSH] OR “Indigenous Peoples”[MeSH] OR “Minority Groups”[Mesh] OR “ethnic*”[Title/Abstract] OR “cultural divers*”[Title/Abstract] OR “cross-cultur*”[Title/Abstract] OR “cultural characteristics”[Title/Abstract:~3] OR “cultural characteristic”[Title/Abstract:~3] OR “cultural diversity”[Title/Abstract:~3] OR “culturally diverse”[Title/Abstract:~3] OR “linguistic diversity”[Title/Abstract:~3] OR “linguistically diverse”[Title/Abstract:~3] OR “CALD”[Title/Abstract] OR “minority group*”[Title/Abstract] OR “racial*”[Title/Abstract] OR “race*”[Title/Abstract] OR “Indigenous”[ Title/Abstract] |
| 4 | #1 AND #2 AND #3 |

**Annex 3: HRI symptoms included in questionnaire per study**

| **#** | **Reference** | **Time frame symptoms^#^** | **Muscle cramps** | **Dizziness** | **Nausea** | **Confusion** | **Dry skin or skin rash** | **Fainting** | **Headache** | **Sweating** | **Weakness** |
| --- | --- | --- | --- | --- | --- | --- | --- | --- | --- | --- | --- |
| 1 | Albu, I., 2023 | Short term | X | X | X | X |  | X | X | X |  |
| 2 | Arcury, T. A., 2015 | Long term | X | X | X | X | X |  |  |  |  |
| 3 | Arcury, T. A., 2020 | Long term | X | X | X | X | X | X |  |  |  |
| 4 | Bethel, J.W., 2014 | Short term | X | X | X | X | X | X | X | X | X |
| 5 | Boonruksa, P., 2020 | Long term | X | X | X |  | X | X | X | X | X |
| 6 | Culp, K., 2019 | Short term | X | X | X | X |  |  |  |  |  |
| 7 | Fleischer, N. L., 2013 | Short term | X | X | X | X | X | X | X |  |  |
| 8 | Gelaye, K.A., 2021 | Short term | X | X |  | X | X | X | X | X | X |
| 9 | Kearney, G. D., 2016 | Short term | X | X | X | X | X | X | X | X | X |
| 10 | McQueen, S.L., 2012 | Short term | X | X | X |  |  |  | X |  |  |
| 11 | Mirabelli, M. C., 2010 | Long term |  |  |  |  |  |  |  |  |  |
| 12 | Odame, E.A., 2019* | Short term |  |  |  |  |  |  |  |  |  |
| 13 | Quiller, G., 2017 | Short term |  | X |  |  |  |  |  | X |  |
| 14 | Riccò, M., 2019* | Long term |  |  |  |  |  |  |  |  |  |
| 15 | Smith, D. J., 2021 | Short term | X | X | X | X |  | X | X | X |  |
| 16 | Spector, J., 2015 | Short term | X | X | X |  | X | X | X | X |  |
| 17 | Villanueva-Gómez, R., 2023 | Long term |  |  |  |  |  |  | X | X |  |

X: Symptom reported in study. | *No specific symptoms reported, only number of symptoms. | **^#^**Referring to the period of time for which participants were asked about symptoms. Long term: In the current season – ever; Short term: During the current working day – in the past week.

**Annex 4: Heat-related illness symptoms in migrant farmworkers – US and Canada only**

|  |  | **Total - US** | | | |  | **Internal migrants - US** | | | |
| --- | --- | --- | --- | --- | --- | --- | --- | --- | --- | --- |
|  | Sample size | I^2^ | Pooled proportion | # Studies | Prediction interval | Sample size | I^2^ | Pooled proportion | # Studies | Prediction interval |
| At least 1 symptom | 1682 | 96% | **0.52** (0.39-0.65) | 12 | 0.09-0.94 | 1413 | 97% | **0.53** (0.36-0.69) | 8 | 0.04-0.98 |
| At least 3 symptoms | 661 | 96% | **0.13** (0.02-0.32) | 4 | 0.00-0.99 | 562 | 94% | **0.18** (0.05-0.37) | 3 | 0.00-1.00 |
| Headache | 986 | 93% | **0.33** (0.21-0.45) | 8 | 0.02-0.75 | 703 | 92% | **0.35** (0.22-0.50) | 4 | 0.00-0.95 |
| Muscle cramps | 1289 | 94% | **0.17** (0.09-0.27) | 10 | 0.00-0.59 | 840 | 88% | **0.24** (0.16-0.34) | 6 | 0.02-0.61 |
| Dizziness | 1333 | 90% | **0.12** (0.06-0.18) | 11 | 0.00-0.40 | 840 | 84% | **0.15** (0.09-0.22) | 6 | 0.00-0.43 |
| Nausea | 1289 | 84% | **0.07** (0.03-0.11) | 10 | 0.00-0.24 | 840 | 84% | **0.07** (0.03-0.13) | 6 | 0.00-0.32 |
| Confusion | 1114 | 88% | **0.05** (0.02-0.10) | 8 | 0.00-0.27 | 840 | 90% | **0.05** (0.01-0.12) | 6 | 0.00-0.38 |
| Dry skin or skin rash | 897 | 97% | **0.17** (0.05-0.33) | 6 | 0.00-0.82 | 780 | 96% | **0.22** (0.09-0.40) | 5 | 0.00-0.89 |
| Fainting | 955 | 70% | **0.01** (0.00-0.03) | 7 | 0.00-0.09 | 739 | 51% | **0.02** (0.00-0.04) | 5 | 0.00-0.10 |
| Sweating | 543 | 74% | **0.40** (0.31-0.49) | 7 | 0.16-0.67 | 301 | 57% | **0.45** (0.36-0.54) | 3 | 0.00-1.00 |
| Weakness | - | - | - | 2 | - | - | - | - | 2 | - |

**Annex 5. Critical appraisal of included studies using the JBL prevalence checklist**

| **Reference** | 1. Appropriate sample frame | 2. Appropriate sampling participants^a^ | 3. Adequate sample size^b^ | 4. Subjects / setting described in detail | 5. Data analysis with sufficient coverage sample | 6. Valid methods for identification condition^c^ | 7. Condition measured in reliable way | 8. Appropriate statistical analysis | 9. Adequate response rate | Overall appraisal |
| --- | --- | --- | --- | --- | --- | --- | --- | --- | --- | --- |
| **Weighting** | 1 | 2 | 2 | 1 | 1 | 2 | 2 | 1 | 2 |  |
| Albu 2023 | Yes | No | Yes | Yes | Yes | Unclear | Unclear | Yes | Yes | 8 |
| Arcury 2015 | Yes | Yes | Yes | Yes | Yes | Yes | Yes | Yes | No | 12 |
| Arcury 2020 | Yes | Yes | No | Yes | Yes | Yes | Yes | Yes | No | 10 |
| Betel 2014 | Yes | Yes | Yes | Yes | Yes | Yes | Unclear | Yes | Unclear | 10 |
| Boonruksa 2020 | Yes | Unclear | No | Yes | Yes | Yes | Unclear | Yes | Unclear | 6 |
| Culp 2019 | Yes | Yes | Yes | Yes | Yes | Yes | Yes | Yes | Unclear | 12 |
| Fleischer 2013 | Yes | Yes | Yes | Yes | Yes | Yes | Yes | Yes | No | 12 |
| Gelaye 2021 | Yes | Yes | Yes | Yes | Yes | Yes | Yes | Yes | Yes | 14 |
| Kearney 2016 | Yes | Yes | Yes | Yes | Yes | Yes | Yes | Yes | Yes | 14 |
| Mcqueen 2012 | Yes | Yes | No | Yes | Yes | Unclear | Unclear | Yes | Unclear | 6 |
| Mirabelli 2010 | Yes | Yes | Yes | Yes | Yes | Unclear | Unclear | Yes | No | 8 |
| Odame 2019 | Yes | Yes | Yes | Yes | Yes | Yes | Yes | Yes | Unclear | 12 |
| Quiller 2017 | Yes | Unclear | No | Yes | Yes | Unclear | Unclear | Yes | Unclear | 4 |
| Riccò 2019 | No | Unclear | No | Yes | Yes | Yes | Unclear | Yes | Yes | 7 |
| Smith 2021 | Yes | No | No | Yes | Yes | Yes | Unclear | Yes | Yes | 8 |
| Sector 2015 | Yes | No | Yes | Yes | Yes | Yes | Unclear | Yes | Unclear | 8 |
| Vilanueva-gómez 2023 | Yes | Yes | No | Yes | Yes | Unclear | Unclear | Yes | No | 6 |

When assessing the included studies, the reviewers only focused on data that was included in this meta-analysis and other data presented in the papers were not assessed. The option “unclear” was selected when information was not provided in the paper, while “no” was selected when information was provided but did not meet the criteria. | ^a^As random sampling is not possible for this type of research, sampling was considered appropriate when all workers at the data collection sites (e.g., farm, camp, healthcare facility) were invited for participation. | ^b^Using the sample size calculation (n= Z2P(1-P)) it was calculated that 100 was an adequate sample size and a deviation of ≤5% from this number was considered acceptable. | ^c^As there is no validated tool / questionnaire for measuring HRI we considered methods valid if they were based on existing literature and / or piloted and validated for internal validity.

**Annex 6: Sensitivity analysis – only studies with a score ≥8 on the JBL prevalence checklist**

|  | **Full sample (n=17)** | | | | | **Studies with ≥8 score (n=12)** | | | | |
| --- | --- | --- | --- | --- | --- | --- | --- | --- | --- | --- |
|  | Sample size | I^2^ | Pooled proportion | # Studies | Prediction interval | Sample size | I^2^ | Pooled proportion | # Studies | Prediction interval |
| At least 1 symptom | 1682 | 96% | **0.52**  (0.39-0.65) | 12 | 0.09-0.94 | 1629 | 97% | **0.50**  (0.37-0.64) | 10 | 0.07-0.94 |
| At least 3 symptoms | 1622 | 99% | **0.21**  (0.04-0.44) | 6 | 0.00-0.97 | 1611 | 99% | **0.21**  (0.04-0.46) | 5 | 0.00-0.97 |
| Headache | 2026 | 92% | **0.36**  (0.27-0.45) | 10 | 0.10-0.67 | 1842 | 94% | **0.30**  (0.21-0.40) | 7 | 0.05-0.65 |
| Muscle cramps | 2329 | 95% | **0.20**  (0.13-0.28) | 12 | 0.01-0.55 | 2154 | 94% | **0.16**  (0.10-0.24) | 10 | 0.00-0.50 |
| Dizziness | 2373 | 97% | **0.15**  (0.08-0.26) | 13 | 0.00-0.63 | 2154 | 97% | **0.14**  (0.05-0.25) | 10 | 0.00-0.64 |
| Nausea | 1379 | 83% | **0.07**  (0.04-0.11) | 11 | 0.00-0.24 | 1204 | 84% | **0.06**  (0.03-0.10) | 9 | 0.00-0.23 |
| Confusion | 2064 | 99% | **0.08**  (0.00-0.22) | 9 | 0.00-0.74 | 2064 | 99% | **0.08**  (0.00-0.22) | 9 | 0.00-0.72 |
| Dry skin or skin rash | 1937 | 97% | **0.24**  (0.13-0.38) | 8 | 0.00-0.76 | 1847 | 97% | **0.21** (0.09-0.35) | 7 | 0.00-0.74 |
| Fainting | 1995 | 94% | **0.02**  (0.00-0.07) | 9 | 0.00-0.26 | 1905 | 95% | **0.02**  (0.00-0.06) | 8 | 0.00-0.27 |
| Sweating | 1583 | 92% | **0.46**  (0.35-0.57) | 9 | 0.13-0.81 | 1440 | 79% | **0.39**  (0.32-0.46) | 6 | 0.19-0.61 |
| Weakness | 1281 | 99% | **0.45**  (0.16-0.76) | 4 | 0.00-1.00 | 1191 | 99% | **0.28**  (0.05-0.62) | 3 | 0.00-1.00 |
